# Commentary on the adoption of a test-based versus syndromic-based approach to outbreak declaration and management in hospital and institutional settings

**DOI:** 10.14745/ccdr.v50i34a03

**Published:** 2024-04-30

**Authors:** Patrick Galange, Richard Mather, Barbara Yaffe, Michael Whelan, Michelle Murti

**Affiliations:** 1 University of Toronto, Dalla Lana School of Public Health, Toronto, ON; 2 Public Health Ontario, Toronto, ON; 3 Queen’s University, Department of Family Medicine, Kingston, ON; 4 Office of the Chief Medical Officer of Health, Ministry of Health, Toronto, ON

**Keywords:** respiratory infections, outbreak, testing, syndromic surveillance, institutions

## Abstract

At present, Ontario, like most other jurisdictions in Canada, uses a syndromic-based surveillance definition for acute respiratory infection (ARI) outbreaks in institutions and public hospitals. Confirmed outbreaks are defined as either two or more ARIs in 48 hours with any common epidemiological link and at least one that is laboratory-confirmed; or three cases of ARIs occurring within 48 hours with any common epidemiological link, and not necessarily with lab confirmation. However, with the adoption of broader test-based approaches for sick patients/residents throughout the pandemic, new challenges have surfaced regarding the declaration and management of ARI outbreaks with a variety of scenarios in respiratory testing results. Decisions, including the determination of epidemiological linkage when there are discordant/negative test results, have become more complicated with the addition of virus-specific test results for every sick individual. The ARI outbreak case definition and management guidance was updated in 2018. The purpose of this commentary is to highlight epidemiological trends in ARI outbreaks in Ontario over the 2022–2023 season compared to the 2018–2019 and 2019–2020 pre-pandemic seasons. This is followed by a discussion around some of the benefits and challenges of implementing a test-based versus syndromic-based approach to ARI outbreaks.

## Introduction

Respiratory infection (RI) outbreaks in hospitals and congregate care settings are common and can have serious implications ((1,2)). Impacts include increased morbidity and mortality, stresses on human health resources, psychological effects of isolation on patients/residents and their families, as well as higher healthcare costs. Not surprisingly, these factors can place a significant burden on an already strained healthcare system ((1)). Effective outbreak identification and management is essential to keep residents and staff safe while maintaining quality of life.

At present, Ontario, like most other jurisdictions in Canada, uses a syndromic-based surveillance definition for RI outbreaks in institutions and public hospitals ((3–6)). Confirmed outbreaks are defined as either two or more acute respiratory infections (ARIs) within 48 hours with any common epidemiological link and at least one that is laboratory-confirmed; or three cases of ARI occurring within 48 hours with any common epidemiological link, and not necessarily with lab confirmation ((3)). However, with the adoption of broader test-based approaches for sick patients/residents throughout the pandemic, new challenges have surfaced regarding the declaration and management of RI outbreaks with a variety of scenarios in respiratory testing results. Decisions, including the determination of epidemiological linkage when there are discordant/negative test results, outbreak attribution of cases, and declaring multiple concurrent outbreaks have become more complicated with the addition of virus-specific test results for every sick individual. The RI outbreak case definition and management guidance was updated in 2018. The purpose of this commentary is to highlight epidemiological trends in RI outbreaks in Ontario over the 2022–2023 season compared to the 2018–2019 and 2019–2020 pre-pandemic seasons. This is followed by a discussion of issues and gaps in current outbreak management, with particular attention to considerations for syndromic-based versus test-based approaches to declaring and managing RI outbreaks in hospitals as well as congregate settings, specifically long-term care and retirement homes.

## Results

### Changes in the 2022–2023 season

There were a number of significant changes to the prevention, declaration, and management of institutional outbreaks throughout the coronavirus disease 2019 (COVID-19) pandemic. These include, among others: enhanced infection prevention and control (IPAC) measures (e.g., universal masking, increased use of alcohol-based hand rub); potential supports available from specialists in infectious diseases, medical microbiology, public health, and IPAC; regular and more frequent testing of staff; extended testing capacity through increased laboratory hours and personnel; and an increase in multi-pathogen testing in 2022–2023, as well as the routine use of rapid antigen tests (RATs) for COVID-19 ((7,^8^)). Taken together, these procedures and protocols may have impacted the overall size, attack rate, duration and/or frequency of RI outbreaks (7).

Of particular interest, an updated approach to testing likely had a significant contribution to the observed trends in mixed and unknown pathogen outbreaks. Historically, only the first four residents with ARI were eligible for multiplex respiratory virus PCR (MRVP) testing by Public Health Ontario’s laboratory, with no routine testing on subsequently sick residents for that outbreak. However, throughout the COVID-19 pandemic, the province implemented routine COVID-19 testing for all symptomatic residents to ensure identification of all cases, but multiplex was still limited to the first four cases. Subsequently, the province expanded eligibility for RI outbreak testing in the fall of 2022, in addition to the first four individuals being eligible for MRVP, all subsequent symptomatic individuals were eligible for COVID-19, influenza, and respiratory syncytial virus (RSV) testing with a rapid turnaround time for results ((9)).

### Respiratory infection outbreaks in the 2018–2020 seasons versus 2022–2023

Publicly available data from the Ontario Respiratory Virus Tool as of February 14, 2024, on RI outbreaks in institutions and public hospitals are summarized in **Table 1** ((10)).

**Table 1:** Reported respiratory infection outbreaks in institutions and public hospitals^a^ and a comparison of the outbreak proportions^b^, for pre-pandemic seasons 2018–2020 versus 2022–2023^c^

Outbreak type	2018–2019	2019–2020	Combined 2018–2020	2022–2023	Uncorrected chi-square	*p*-value (two-tail)
Total outbreaks	1,643	1,018	2,661	1,679	N/A	N/A
Multiple pathogen outbreaks (proportion of total)	8 (0.5%)	53 (5.2%)	61 (2.3%)	135 (8.0%)	78.9	<0.001
Unknown pathogen outbreaks (proportion of total)	796 (48.4%)	353 (34.7%)	1,149 (43.2%)	193 (11.5%)	483.8	<0.001

Abbreviation: N/A, not applicable

^a^ By total, multiple pathogen and unknown pathogen outbreaks

^b^ Proportion of multiple and unknown pathogen outbreaks

^c^ Dates ranges: 2018–2019 (August 26, 2018, to August 24, 2019), 2019–2020 (August 25, 2019, to March 7, 2020), Combined 2018–2020 (up to March 7, 2020, in 2019–2020), 2022–2023 (August 28, 2022, to August 26, 2023)

Note: This table does not include COVID-19 outbreaks, even for multiple pathogen outbreaks and unknown pathogen outbreaks

In comparison to the pre-pandemic 2018–2019 and 2019–2020 (up to March 7, 2020) seasons, in 2022–2023, there was a significant increase in the proportion of outbreaks with “multiple pathogens.” Similarly, in 2022–2023, there was a significant decrease in the proportion of outbreaks with “unknown pathogen” versus the comparable pre-pandemic seasons. An increase in outbreaks involving “multiple pathogens” is clinically relevant due to historically longer median duration of the outbreak when compared to those with only a single pathogen ((1)). Declines in “unknown pathogen” outbreaks are also clinically relevant, as virus-specific interventions (e.g., prophylaxis or application of virus-specific incubation periods when declaring an outbreak over) can be applied when there is a known causative virus.

Enhanced testing is the likely driver for the shift in trends in multiple and unknown pathogen outbreaks. However, enhanced testing may have also resulted in other trends in outbreaks for which data are not publicly available, such as changes to attack rates if inclusion of cases is based on test results instead of symptoms or if increased testing was applied to mildly symptomatic (non-ARI) individuals, and/or changes to outbreak duration if based on last laboratory-confirmed case versus last symptomatic case (7).

## Discussion

Syndromic-based versus test-based respiratory infection outbreak management

The trends in the 2022–2023 season warrant a discussion on the issues and gaps of the current syndromic-based versus test-based outbreak definition and management approaches commonly used in Canada. First, in an ideal scenario, it enables the healthcare system to comprehensively identify and manage causative agents for all sick individuals in the outbreak. While historical multiplex testing of only initial cases in an outbreak (e.g., “first four” testing in Ontario) was sufficient for the majority of outbreaks, the decline in “unknown pathogen” outbreaks in institutions in 2022–2023 suggests that additional testing enables identification of a causative virus in a higher proportion of outbreaks. At an individual resident level, identification of influenza or COVID-19 allows for accurate initiation of oseltamivir or nirmatrelvir/ritonavir, respectively, which are both time-sensitive and life-saving interventions. Likewise, providers can more confidently initiate antiviral prophylaxis to suppress influenza outbreaks and avoid initiation/usage of antiviral prophylaxis for non-influenza outbreaks ((11)).

A second advantage of a test-based approach is the enhanced understanding of RI outbreak epidemiology for future vaccine and therapeutics programs, such as for RSV. For example, testing improves assessment of RSV prevalence in institution populations, to support consideration of an RSV vaccine program with forthcoming RSV vaccines ((12)). Third, existing syndromic-based approaches rely on the definition of ARI for inclusion of cases in an outbreak. Certain populations, such as the elderly, may not present with “classic” ARI symptoms. These cases may be missed if relying on a syndromic-based approach and not tested but would be captured if using a test-based approach ((13)). Of note, this argument becomes less pertinent when applying a more sensitive syndromic case definition, which includes non-respiratory symptoms such as a decrease in function or increased falls.

Conversely, there are also challenges with a test-based approach. First, decision-making is more challenging and time-consuming for outbreak management when performing a test on every symptomatic individual versus presuming their association with the outbreak and managing them accordingly. There may be a delay in initiation of treatment and prophylaxis if the outbreak management team must now wait for individual test results versus treating empirically. Second, although additional testing might optimize resource utilization in the long run, the upfront cost of these tests is not immaterial ((14)). Decision-makers for public health financing need to consider the costs versus benefits of adopting increased use of MRVP and/or COVID-19/influenza/RSV panel testing ((15)). Third, it is unclear if test-based approaches improve key outcomes, such as morbidity and mortality, for outbreaks. However, decreasing the frequency of “multiple pathogen” outbreaks could shorten outbreak duration and lessen restrictions on recreational programs, significantly enhancing residents’ quality of life. Fourth, these proposed changes may actually result in fewer outbreaks reported as having “multiple pathogens.” Rather, these might be considered as multiple concurrent outbreaks, increasing the total number of outbreaks reported. Fifth, this testing method could lead to an overemphasis on test outcomes by staff, overshadowing assessment of the whole patient. Lastly, test-based approaches need to address interpretation of negative test results and situations when individuals are not tested when a specific organism is identified in other patients/residents. For example, consider a situation of two epidemiologically linked patients with ARI, where one tested positive for influenza but the other tested negative. This would technically meet the current Ontario outbreak definition, hinging on the determined strength of the ”epidemiological linkage” of the two cases and presumption that the negative test is a ”false negative,” possibly due to improper technique or timing of specimen collection. Historically, no additional testing would be conducted once the influenza outbreak was declared. It would then trigger initiation of antiviral chemoprophylaxis for the whole outbreak area, and initiation of antiviral treatment for all newly symptomatic individuals ((8)). However, if subsequently sick residents were all tested and also found to be negative (or to have a different virus), the influenza-based outbreak management could be deemed unnecessary and discontinued.

## Conclusion

Respiratory infection outbreaks in hospital and institutional settings are common and can be severe. As a result of the COVID-19 pandemic, there were a number of changes to the identification and management of RI outbreaks in these settings following the last guidance update in 2018 (pre-pandemic) and the 2022–2023 respiratory season in Ontario. This commentary explores the benefits and challenges of adopting a more test-based approach to the outbreak declaration and management compared to one that is syndromic-based. More data and evaluation are needed to further assess whether the use of increased test-based approaches has a meaningful impact on outbreak management outcomes, and if it is cost-effective. For example, it would be important to stratify these impacts across different types of institutional settings, as well as an account for any differences in staff capabilities and patient populations. Ultimately, the exploration of test-based versus traditional syndromic-based methods underscores the need for a nuanced approach that not only enhances outbreak management effectiveness, but also significantly improves the quality of life for residents, resulting in better overall health outcomes.
